# Arrest Chemokines

**DOI:** 10.3389/fimmu.2014.00150

**Published:** 2014-04-04

**Authors:** Klaus Ley

**Affiliations:** ^1^Division of Inflammation Biology, La Jolla Institute for Allergy and Immunology, La Jolla, CA, USA

**Keywords:** chemokine, leukocyte adhesion, integrin, talin, kindlin-3, LFA-1, VLA-4, signal transduction

Chemokines are a large family (~50 members) of chemoattractants that bind to cognate chemokine receptors (~25 known). Leukocytes roll along the vascular endothelium through selectins interacting with their glycoprotein ligands until they encounter a chemokine that stops them in their tracks (1, 2). The fact that chemokines can induce arrest of rolling leukocytes and make them adhere was discovered in the 1990s (3–6), and the term “arrest chemokines” was coined in 2003 (7). Many chemokines including CXCL1, 2, 8, 9, 10, 12, CCL 3, 5, 11, 19, 21, and CX3CL1 have been shown to activate leukocyte integrins and induce arrest, but other chemokines may also have this ability and simply have not been tested in rolling-to-arrest assays. In this Research Topic, 26 authors have contributed 9 articles touching on many of the known arrest chemokines. This Research Topic is aimed at covering the structure, expression, and physiological function of arrest chemokines, the biophysical processes associated with leukocyte arrest, and the molecular mechanisms of rapid leukocyte integrin activation responsible for arrest.

Bongrand’s group has pioneered the study of the biomechanics of cell adhesion for the past 30 years (8). In their contribution to this Research Topic (9), they discuss the finite time required for integrin activation, the nanoscale dynamics of the arrest process, and the contribution of local membrane deformation. They apply this knowledge of the biomechanics of leukocyte arrest to the study of the leukocyte arrest defect seen in patients with leukocyte adhesion deficiency (LAD) type III. In this disorder, the cytoskeletal protein kindlin-3 is not expressed and integrin activation is impaired.

Once rolling leukocytes encounter immobilized or soluble chemokine, a series of signaling events is triggered that ultimately results in integrin activation by conformational extension, affinity increase, and clustering. The proximal signaling is clear: the chemokine binds its G-protein coupled receptor and the Gα subunit dissociates from Gβγ. The distal signaling is also fairly clear: both talin-1 and kindlin-3 bind to the cytoplasmic domain of the β chain of the leukocyte integrin responsible for arrest. But what links the two processes is an area of active investigation. Laudanna and colleagues focus on the roles Rap1 and RhoA, two of many small G proteins found in leukocytes (10).

Another signaling paper in this Research Topic focuses on calcium. Intracellular free calcium rises rapidly when a chemokine binds its receptor, because the dissociated Gβγ subunit of chemokine receptors can trigger calcium release from intracellular stores by activating phospholipase C (PLC)β. It has long been known that arrest is associated with a rise in intracellular free calcium (11), but it is not known whether this is required and if so, for which step in the signaling cascade. Scott Simon’s group has worked on calcium signaling induced by selectin-mediated leukocyte interactions (12). In their contribution to the Research Topic, Simon’s group focuses on the calcium rise that occurs after arrest (13). Their work suggests that elevated intracellular free calcium is required to induce a migratory phenotype in arrested neutrophils.

Rolling leukocytes do not always stop, but may instead slow down considerably. This slower rolling is associated with partial integrin activation to a state that is known as extended. Talin-1 binding to integrin appears to be sufficient for this. However, for arrest to occur, integrin extension appears necessary, but not sufficient: a high affinity conformation of integrin is needed. This last step can be induced by chemokines and requires kindlin-3 (14). Lefort and Ley suggest that talin-1 is required for both integrin extension and high affinity, and kindlin-3 is only required for inducing the high affinity conformation. A competing hypothesis is that kindlin-3 may be involved in integrin clustering (15). More direct evidence in primary leukocytes will be needed to distinguish between these two competing models.

Chigaev and Sklar have pioneered the use of small fluorescent peptides to report the activation of integrins. In their contribution to the Research Topic, they review the insights obtained by this approach with a focus on the αLβ2 integrin LFA-1 expressed by all leukocytes and α4β1 integrin expressed by monocytes and lymphocytes (16).

Among the ~50 chemokines known, only a handful functions as arrest chemokines. One requirement seems to be binding to the endothelial surface, but not all chemokines that bind to the endothelial surface induce arrest. Weber’s group was among the first to describe arrest chemokines (17). In their contribution to this Research Topic, Weber’s group reviews human chemokines and the therapeutic potential of modulating their function (18).

Macrophage inhibitory factor (MIF) is not a classical chemokine, but signals through the chemokine receptor CXCR2 and can activate LFA-1 (19). Bernhagen’s group proposes that MIF binding to CXCR2 initiates a “motility program” in leukocytes. Because CXCR2 is one of the most efficient chemokine receptors triggering arrest, and because it has at least eight known ligands, a separate review in this Research Topic is focused on this one receptor (20). CXCR2 has been targeted by small allosteric inhibitors, and some of these show promise in clinical trials, which is the focus of the contribution by Zarbock’s group (20).

Some chemokine receptors do not signal through dissociation of Gα from Gβγ. Initially, these receptors including Duffy antigen receptor for chemokines (DARC) and D6 were called decoy receptors, because they were thought to sequester chemokines and prevent them from having effects. In recent years, it has become clear that these receptors have important functions in transporting chemokines across endothelial cells. In their contribution to this Research Topic, Antal Rot’s group focuses on the role of DARC in this process. In fact, DARC may be a receptor that positions chemokines correctly on the endothelial surface to fulfill their arrest function (21).

Although progress on arrest chemokine function over the last 20 years has been remarkable, many aspects still require more work. It is controversial whether arrest chemokines and their receptors are monomers, homodimers, or heterodimers. It remains unknown how calcium signaling may be involved in integrin activation. We can expect that the exact function of talin-1 and kindlin-3 in integrin activation will be discovered through novel structure–function and live cell imaging approaches. An exciting prospect of more research aimed at understanding arrest chemokines is that their manipulation may have therapeutic potential in inflammatory diseases.
